# Bibliometric and Visualization‐Based Analysis of Global Research Trends on Platelet‐Rich Plasma in Skin Aging

**DOI:** 10.1111/jocd.71133

**Published:** 2026-08-17

**Authors:** Yujun Jiang, Yan Teng, Yang Ding, Jing Luo, Qiong Gao, Xiaohua Tao, Jie Chen

**Affiliations:** ^1^ Center for Plastic & Reconstructive Surgery, Department of Dermatology Zhejiang Provincial People's Hospital, Affiliated People's Hospital, Hangzhou Medical College Hangzhou People's Republic of China; ^2^ Zhuji People's Hospital of Zhejiang Province Zhuji People's Republic of China

**Keywords:** bibliometrics, CiteSpace, photoaging, platelet‐rich plasma, PRP, skin aging, VOSviewer

## Abstract

**Background:**

Platelet‐rich plasma (PRP), as an autologous concentrate enriched with platelets and growth factors, plays a critical role in regulating skin cell function, stimulating collagen synthesis, and mediating tissue repair processes related to skin aging, including extracellular matrix remodeling, inflammatory modulation, and angiogenesis. This study aimed to provide a comprehensive bibliometric overview of the current research landscape, key trends, and emerging hotspots in PRP research on skin aging.

**Methods:**

Bibliometric analysis was performed using CiteSpace (Version 6.4.R1), VOSviewer (Version 1.6.20), Tableau (2025.2), and Excel (2021). Data were retrieved from the Science Citation Index Expanded of the Web of Science Core Collection, covering publications from 2014 to 2026. The analysis encompassed publication trends, country/region distribution, institutional contributions, core journals, influential authors, citation patterns, and research hotspots.

**Results:**

A total of 310 relevant publications (original articles and reviews) were identified, with a marked upward trend in publications since 2018 and a peak of 47 papers in 2025. The USA contributed the largest number of publications (79), followed by China (54) and Italy (40). Iran University of Medical Sciences (Iran) was the most productive institution, while Journal of Cosmetic Dermatology published the highest number of articles in this field. Behrangi E and Zare S were the most productive authors, and Hu L's 2018 article in Theranostics received the highest local citation score (476). Key research hotspots included “platelet‐rich plasma,” “skin rejuvenation,” “wound healing,” and “growth factors,” while emerging themes such as “efficacy,” “safety,” “clinical trial,” and “double‐blind” reflected a growing emphasis on clinical validation.

**Conclusion:**

Research on PRP in skin aging has grown rapidly, with the USA, China, and Italy leading in output and influence. The field is transitioning from exploratory investigations to evidence‐based clinical validation, with increasing focus on randomized controlled trials, combination therapies (hyaluronic acid, laser, fillers), and mechanistic studies (oxidative stress, apoptosis). Future efforts should emphasize strengthening international collaboration—particularly bridging regionally clustered networks, enhancing research quality and translational impact, and expanding multi‐database analyses to advance safe, effective PRP‐based interventions for skin aging globally.

## 1 Introduction

Skin aging is a complex biological process that affects individuals worldwide, characterized by visible structural and functional changes, such as wrinkles, sagging, dryness, reduced elasticity, and impaired wound healing capacity [1]. It is influenced by multiple factors, including intrinsic genetic factors, hormonal changes, and extrinsic stressors like ultraviolet radiation, pollution, smoking, and poor nutrition [2, 3, 4, 5]. The molecular mechanisms underlying skin aging involve oxidative stress, chronic inflammation, telomere shortening, and extracellular matrix degradation—particularly the progressive loss of collagen and elastin fibers—which have been extensively investigated [6]. In recent years, platelet‐rich plasma (PRP), an autologous concentrate derived from whole blood containing high concentrations of platelets and associated growth factors, has emerged as a promising therapeutic modality in regenerative medicine and aesthetic dermatology [7]. Upon activation, platelets release bioactive molecules, including platelet‐derived growth factor (PDGF), transforming growth factor‐beta (TGF‐β), vascular endothelial growth factor (VEGF), and epidermal growth factor (EGF), which mediate various physiological processes such as cell proliferation, angiogenesis, and tissue repair [8].

Accumulating evidence suggests that PRP contributes significantly to interventions for skin aging. PRP modulates skin cell function, stimulates fibroblast activity and collagen synthesis, regulates the inflammatory microenvironment, and influences extracellular matrix remodeling [9]. Numerous preclinical studies have demonstrated that PRP promotes skin cell regeneration, enhances dermal thickness, reduces oxidative damage, and alleviates inflammation. Moreover, a growing number of clinical trials and aesthetic procedures have explored PRP in anti‐aging therapies, with promising results in improving skin texture, elasticity, and overall appearance, particularly in facial rejuvenation, hair restoration, and scar treatment. Given the increasing interest in PRP and its relevance to skin aging research, a quantitative analysis of research progress, key findings, and future directions is essential.

Bibliometrics, a valuable tool in library and information science, enables systematic analysis of publication patterns, co‐authorship networks, citation relationships, institutional collaborations, and keyword trends [10]. Software tools like CiteSpace and VOSviewer facilitate the visualization and interpretation of bibliometric data, helping researchers uncover latent knowledge structures, identify research hotspots, and track evolving research landscapes [11]. Bibliometric analysis has been widely applied across various medical fields, including stem cell therapy, tissue engineering, and dermatological interventions. However, comprehensive bibliometric studies specifically focusing on PRP in skin aging remain scarce. Therefore, this study aimed to systematically assess the research status, productivity patterns, international collaborations, hotspots, and emerging frontiers of PRP, providing evidence‐based insights to guide future research and clinical applications in this rapidly evolving field.

## 2 Method

### 2.1 Database and Extraction

**FIGURE 1:**
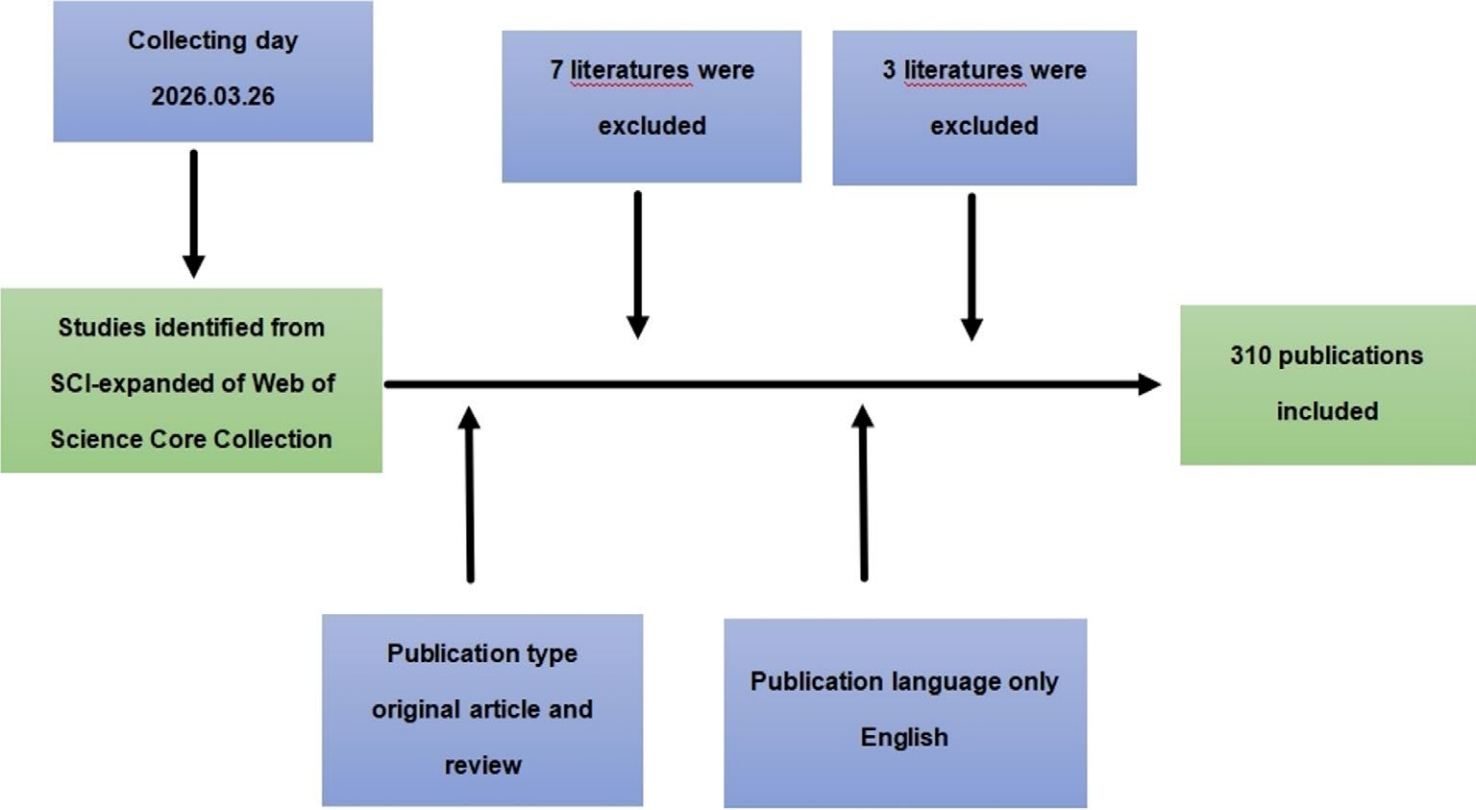
Flowchart outlining the data gathering process for PRP and skin aging research field.

A systematic search strategy was developed and executed in the Web of Science Core Collection. To minimize bias from dynamic database updates, all retrieval procedures and data exports were completed in a single session on March 3, 2026. The search was restricted to literature examining the relationship between PRP and skin aging, using the following query: TS = (“Platelet‐rich plasma” OR “platelet rich plasma” OR “PRP”) AND TS = (“skin aging” OR “photoaging” OR “solar aging” OR “skin wrinkling”), yielding an initial pool of 320 records. To reduce potential bias in subsequent analyses, the following inclusion criteria were applied: (i) publication date within the specified timeframe; (ii) document type restricted to original research articles and reviews (conference abstracts, letters, and book chapters were omitted to maintain analytical rigor); (iii) English language only to ensure interpretive uniformity. After systematic deduplication and thorough eligibility assessment, 310 publications were selected for comprehensive bibliometric evaluation. Data extraction was performed independently by two reviewers to ensure reliability. The complete selection process is depicted in Figure 1.

### 2.2 Data Analysis

The full dataset—including full records and cited references (titles, keywords, authors, affiliations, countries, journals, and reference lists)—was exported from the Web of Science in plain‐text (.txt) format. Exported files were manually screened to remove any anomalies that could compromise accuracy. The cleaned dataset was then imported into CiteSpace (v6.4.R1), VOSviewer (v1.6.20), Tableau (2025.2), and Excel (2021) for subsequent bibliometric analyses.

## 3 Results

### 3.1 Publishing Trend Analysis

**FIGURE 2:**
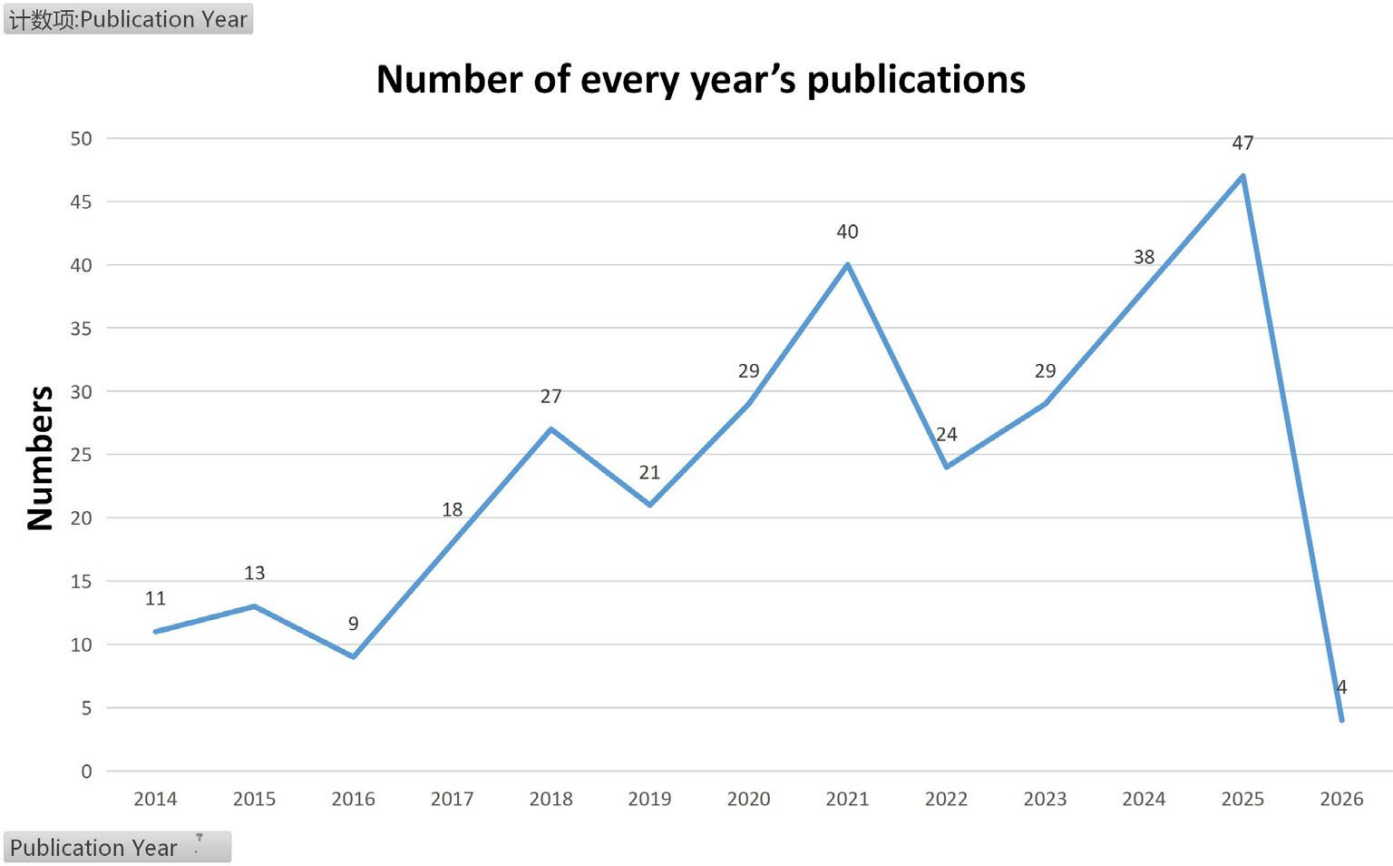
Annual trends in global publication output in the PRP and skin aging research field.

Figure 2 presents the annual number of publications in the PRP and skin aging research field from 2014 to 2026. Overall, annual publication output showed an upward trend over this period, increasing from 11 papers in 2014 to 47 papers in 2025, indicating steady expansion in research activity. The trend exhibited moderate fluctuations: after a slight rise from 2014 (11 papers) to 2015 (13 papers), output declined to a low of 9 papers in 2016, followed by a consistent increase to 27 papers in 2018. Subsequently, the number dropped temporarily to 21 papers in 2019 before surging to a period high of 40 papers in 2021. It then fell to 24 papers in 2022, rebounded to 29 papers in 2023, rose to 38 papers in 2024, and peaked at 47 papers in 2025.

Notably, only four papers were recorded for 2026 at the time of the search (March 14, 2026), as the analysis captured only publications released between January 1 and March 14, 2026. These trends indicate that the PRP–skin aging field has attracted sustained academic attention over the past decade, with a growing number of researchers engaging in related studies. The fluctuations reflect dynamic shifts in research focus, while the overall upward trajectory underscores continuous expansion in research scale and academic influence.

### 3.2 Countries and Contribution

**FIGURE 3:**
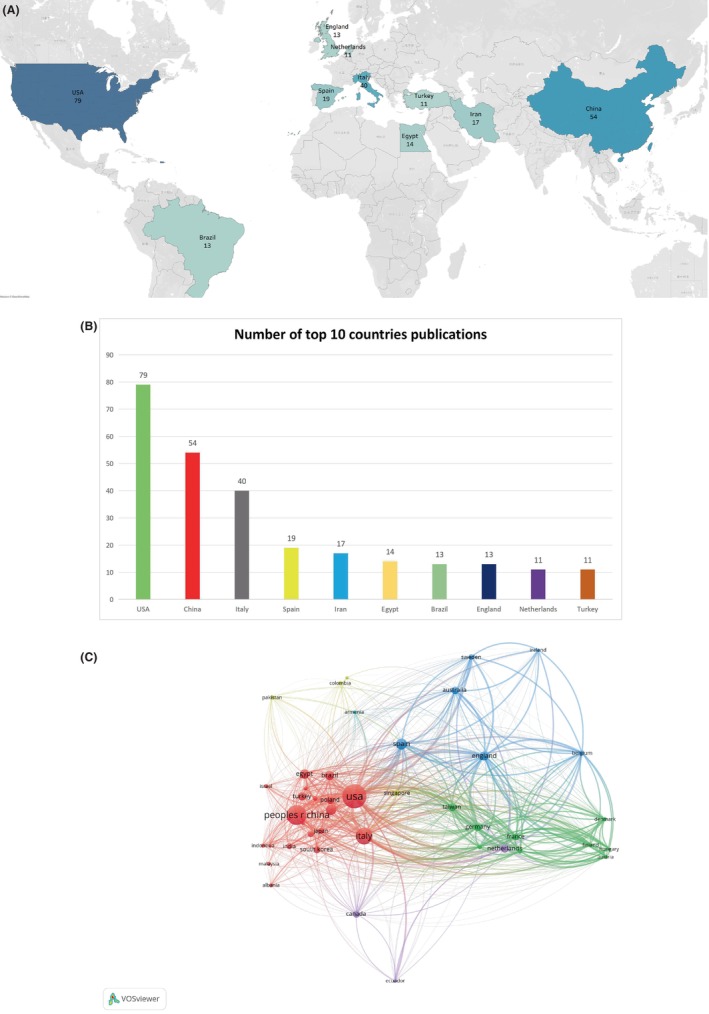
Analysis of the distribution of countries or regions. (A) Geographical distribution of the countries/regions in terms of publications. Country name and publication numbers are shown on the map. (B) Histogram of the top 10 countries by number of publications. (C) Network map of international collaboration.

Numerous countries and regions worldwide have contributed to research on PRP and skin aging, with the global distribution of publications visualized in the world map (Figure 3A). The top‐performing countries have demonstrated substantial research output, reflecting widespread academic interest in this interdisciplinary field.

To examine international collaboration patterns, we constructed a country‐level co‐authorship network using VOSviewer (Figure 3C), in which node size corresponds to total publication volume and connecting lines denote collaborative ties. The network reveals distinct clustering: The U.S. (the largest node, signifying the highest publication output) serves as a central hub with extensive, intensive collaborations with nations like China, Italy, and Brazil; European countries (including England, Spain, the Netherlands, and Nordic states such as Sweden and Ireland) form tightly linked cooperative clusters; and China acts as another key hub, maintaining robust collaborative connections with major global research countries.

As shown in Table 1 and visualized in Figure 3B, the USA ranks first with 79 papers (31.19% of the total publications), coupled with its high total citations (2328) and a citation rate of 29.47 per publication, underscoring its dominant position. China ranks second with 54 papers (17.66%) and 1318 total citations (24.41 per publication). Italy ranks third with 40 papers (16.19%), 1208 total citations, and 30.2 citations per publication. Spain (19 papers), Iran (17 papers), and Egypt (14 papers) rank fourth through sixth, respectively. Notably, England has the highest citations per publication (37.92) despite ranking eighth in total output (13 papers), while Italy (30.2) and Egypt (30.57) also demonstrate strong citation impact. Other notable contributors include Brazil and England (13 papers each), and the Netherlands and Turkey (11 papers each), ranking seventh through tenth, respectively. These findings indicate that the PRP‐skin aging research field has attracted extensive global participation, with the USA and China as core contributors—together accounting for nearly 49% of total publications. The co‐authorship network further reveals active international collaboration, although clustered cooperation patterns (e.g., dense links among European nations) are apparent.

**TABLE 1:** The top 10 productive countries/regions in PRP in the skin aging research field.

Rank	Countries	Publications	Proportion of publications	Citations	Citations per publication
1	USA	79	31.19%	2328	29.46835443
2	China	54	17.66%	1318	24.40740741
3	Italy	40	16.19%	1208	30.2
4	Spain	19	7.12%	531	27.94736842
5	Iran	17	6.26%	467	27.47058824
6	Egypt	14	5.73%	428	30.57142857
7	Brazil	13	2.37%	177	13.61538462
8	England	13	6.61%	493	37.92307692
9	the Netherlands	11	3.03%	226	20.54545455
10	Turkey	11	3.85%	287	26.09090909

### 3.3 Institutions

**FIGURE 4:**
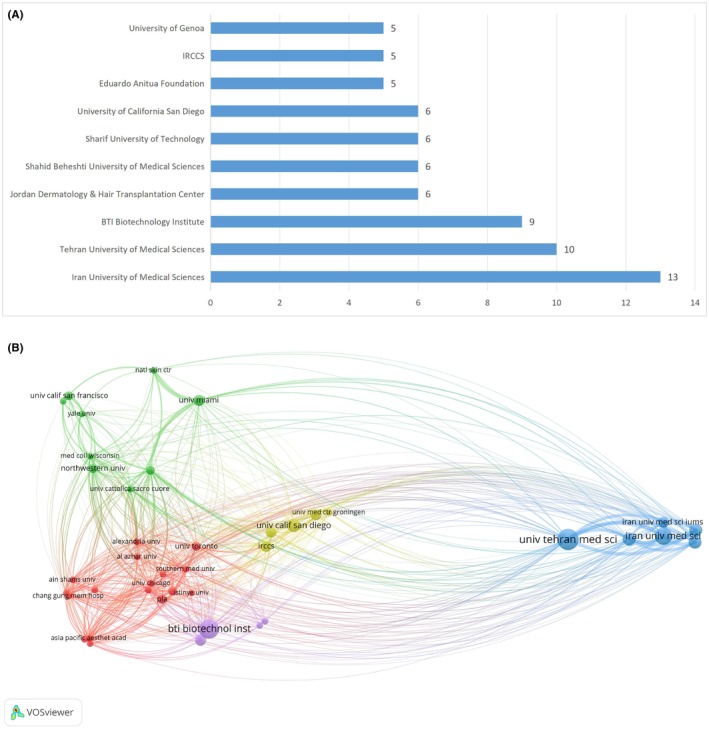
Analysis of institutions. (A) Histogram of the top 10 institutions by number of publications. (B) The co‐occurrence map of institutions depicts the relationships between them. The size of the nodes corresponds to the number of articles associated with each institution; the thickness of the curves indicates the strength of collaboration. The colors used in the map distinguish various collaboration groups.

A total of 622 institutions worldwide have contributed to research on PRP and skin aging, with the top 10 most productive institutions (detailed in Table 2) serving as core contributors and accounting for a substantial share of the total publications. As shown in Figure 4A, the ranking of these top 10 institutions by publication volume reveals a distinct regional distribution: Iran leads with 4 institutions, followed by Spain and Italy each with 2, and the United States, and Jordan each contributing 1—underscoring the emerging prominence of Iranian research centers in this field. Among them, Iran University of Medical Sciences ranks first with 13 papers, 133 total citations, and a citation‐per‐publication ratio of 10.23. Tehran University of Medical Sciences (Iran) follows closely with 10 publications, 173 total citations, and 17.3 citations per paper. BTI Biotechnology Institute ranks third with 9 publications and 153 total citations (17 citations per paper).

**TABLE 2:** The top 10 productive institutions within PRP in skin aging research field.

Rank	Institutions	Publications	Citations	Country	Citations per publication
1	Iran University of Medical Sciences	13	133	Iran	10.23
2	Tehran University of Medical Sciences	10	133	Iran	13.3
3	BTI Biotecnology Institute	9	153	Spain	17
4	Jordan Dermatology & Hair Transplantation Center	6	42	Jordan	7
5	Shahid Beheshti University of Medical Sciences	6	33	Iran	5.5
6	Sharif University of Technology	6	33	Iran	5.5
7	University of California San Diego	6	332	United States	55.33333333
8	Eduardo Anitua Foundation	5	97	Spain	19.4
9	IRCCS	5	184	Italy	36.8
10	University of Genoa	5	140	Italy	28

In terms of citation impact per publication, the University of California San Diego (United States) stands out with an impressive ratio of 55.33 (6 papers, 332 total citations), followed by IRCCS (Italy) with 36.8 citations per paper (5 papers, 184 total citations) and Eduardo Anitua Foundation (Spain) with 19.4 citations per paper (5 papers, 97 total citations). Other notable institutions include Jordan Dermatology & Hair Transplantation Center (6 papers, 42 total citations, 7 citations per paper), Shahid Beheshti University of Medical Sciences (6 papers, 33 total citations, 5.5 citations per paper), and Sharif University of Technology (6 papers, 33 total citations, 5.5 citations per paper). Collectively, these top institutions maintain steady publication output and display varying degrees of academic influence, jointly driving the depth and advancement of PRP and skin aging research.

To further explore collaborative dynamics among institutions, we analyzed the institutional co‐authorship network generated via VOSviewer (Figure 4B). In this visualization, each node represents an institution, with node size roughly corresponding to publication volume, and connecting lines indicating collaborative relationships—clustered color groups denote closely linked institutional alliances. Notably, Iranian institutions form a distinct cluster on the right side of the network, with key hubs including Tehran University of Medical Sciences, and Iran University of Medical Sciences, which exhibit dense interconnections and constitute a tightly knit domestic research alliance. Meanwhile, U.S. institutions—including the University of California San Diego, University of Miami, Northwestern University, and Yale University—form another collaborative cluster on the left, with extensive ties to institutions such as the National Skin Centre and University of California San Francisco. European institutions—including IRCCS (Italy), University Medical Center Groningen (Netherlands), and BTI Biotechnology Institute (Spain)—occupy central positions in the network, serving as bridges connecting the Iranian and U.S. clusters. Additionally, Middle Eastern institutions such as Alexandria University, Al‐Azhar University, and Ain Shams University (Egypt) form a separate cluster with strong regional ties, while Asia‐Pacific institutions like Chang Gung Memorial Hospital (Taiwan) maintain selective connections to the core network. Overall, this network highlights the multi‐centric collaborative structure, with Iranian institutions demonstrating strong domestic partnerships, U.S. institutions maintaining cross‐regional linkages, and European centers facilitating global integration—suggesting a relatively balanced yet regionally clustered global research ecosystem that would benefit from enhanced inter‐regional collaboration to further advance the field.

### 3.4 Journals and Cited Journals

**FIGURE 5:**
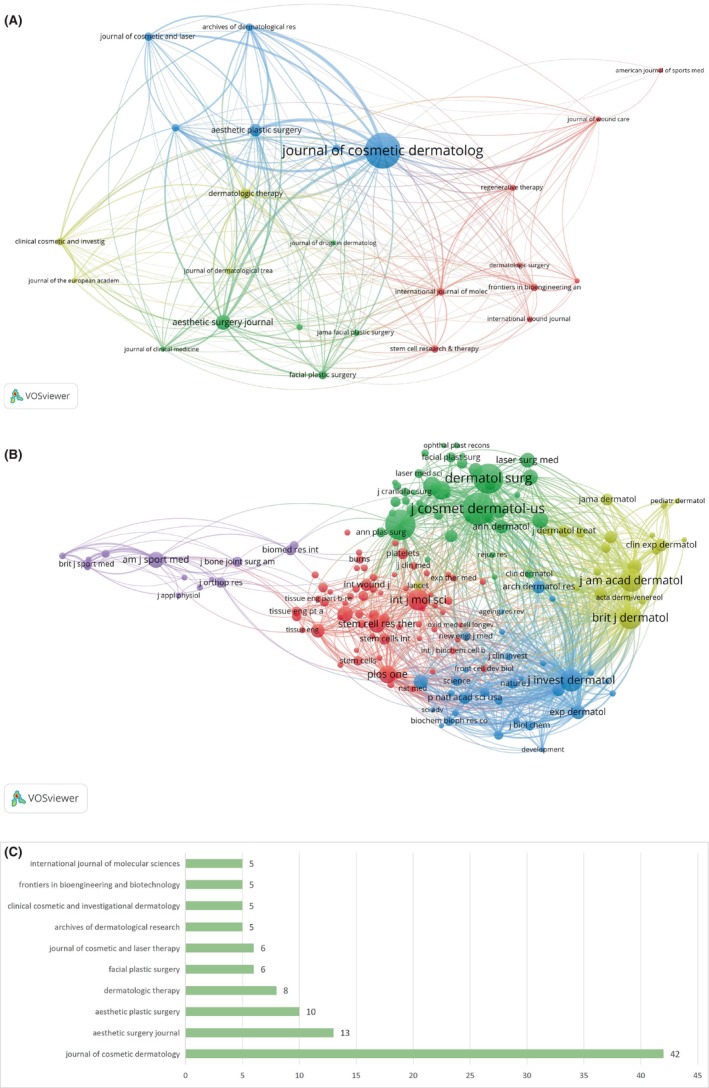
Journals and co‐cited journals analysis. (A) The bibliographic coupling network of journals related to PRP–skin aging. Each circle in the figure represents a journal, and the size of the circle indicates the number of publications in that journal. (B) The co‐citation network visualization of journals with a minimum of 10 citations. The size of the circle indicates the number of citations for that journal. (C) Histogram of the top 10 journals by number of publications.

To date, publications on PRP and skin aging have appeared across a wide array of academic journals, reflecting the interdisciplinary nature of this field. Table 3 and Figure 5C present the top 10 core journals in terms of publication output (NP), providing insights into the most productive and influential periodicals in this domain. Among these leading journals, the *Journal of Cosmetic Dermatology* ranks first with 42 publications, 733 total citations (TC), and an impact factor (IF2025) of 2.5, underscoring its role as a key platform for research bridging PRP therapy and aesthetic dermatology. Following closely is the *Aesthetic Surgery Journal*, with 13 publications, 408 total citations, and an IF2025 of 3.9, while *Aesthetic Plastic Surgery* ranks third with 10 publications, 130 total citations, and an IF2025 of 2.8, highlighting its focus on the translational applications of PRP in cosmetic procedures.

**TABLE 3:** The core journals that published publications in PRP in the skin aging research field.

Rank	Journal	NP	TC	IF2025
1	journal of cosmetic dermatology	42	733	2.5
2	aesthetic surgery journal	13	408	3.9
3	aesthetic plastic surgery	10	130	2.8
4	dermatologic therapy	8	134	3.4
5	facial plastic surgery	6	124	1.4
6	journal of cosmetic and laser therapy	6	116	1.3
7	archives of dermatological research	5	139	2.1
8	clinical cosmetic and investigational dermatology	5	61	2.2
9	frontiers in bioengineering and biotechnology	5	120	4.8
10	international journal of molecular sciences	5	718	4.9

Other prominent journals in the top 10 include *Dermatologic Therapy* (8 publications, 134 total citations, IF2025 = 3.4) and *Facial Plastic Surgery* (6 publications, 124 total citations, IF2025 = 1.4), both reflecting the clinical application of PRP in facial rejuvenation. *The Journal of Cosmetic and Laser Therapy* also contributes 6 publications with 116 total citations (IF2025 = 1.3), emphasizing the combination of PRP with laser modalities. *Archives of Dermatological Research* (5 publications, 139 total citations, IF2025 = 2.1) and C*linical, Cosmetic and Investigational Dermatology* (5 publications, 61 total citations, IF2025 = 2.2) demonstrate the field's connections to fundamental dermatological research and clinical investigation. *Frontiers in Bioengineering and Biotechnology* (5 publications, 120 total citations, IF2025 = 4.8) and the *International Journal of Molecular Sciences* (5 publications, 718 total citations, IF2025 = 4.9) round out the list, each contributing valuable insights to the molecular and biotechnological aspects of PRP research. Notably, the *International Journal of Molecular Sciences* stands out with the highest citation count (718) despite its ninth‐place ranking in total output, indicating strong citation performance. Collectively, these journals span aesthetic dermatology, plastic surgery, molecular biology, and bioengineering, vividly illustrating the interdisciplinary essence of the PRP–skin aging field.

The journal co‐citation network visualized in Figure 5A,B via VOSviewer further reflects the interdisciplinary scope of PRP and skin aging research, encompassing dermatology, aesthetic surgery, regenerative medicine, and molecular biology. In Figure 5A, journals cluster into distinct thematic groups: dermatology‐focused outlets (e.g., *Journal of Cosmetic Dermatology*, *Dermatologic Therapy*, *Archives of Dermatological Research*) form a dense blue‐green cluster at the center, closely linked to aesthetic surgery journals (e.g., *Aesthetic Plastic Surgery*, *Aesthetic Surgery Journal*, *Facial Plastic Surgery*), highlighting the intersection of PRP applications in cosmetic and reconstructive procedures. Meanwhile, regenerative medicine and wound healing journals (e.g., *Stem Cell Research & Therapy*, *Regenerative Therapy*, *Journal of Wound Care, International Wound Journal*) form a distinct red cluster on the right, reflecting the field's connections to tissue repair and cell‐based therapies. A smaller cluster of molecular biology and biotechnology journals (e.g., *Frontiers in Bioengineering and Biotechnology, International Journal of Molecular Sciences*) further underscores the field's scientific foundations.

Figure 5B expands this network to include high‐impact interdisciplinary journals: prominent dermatology outlets (e.g., *Journal of Investigative Dermatology, British Journal of Dermatology, Journal of the American Academy of Dermatology*) anchor a large yellow‐green cluster, while regenerative medicine and stem cell research journals (e.g., *Stem Cells, Tissue Engineering, Biomaterials Research International*) form a central red cluster, with strong links to dermatology and plastic surgery nodes. High‐impact general science and medical journals (e.g., *Nature, Science, New England Journal of Medicine, Lancet, PLOS ONE*) also appear as key connectors, reflecting landmark studies on PRP mechanisms and clinical applications. The dense interconnections between clusters—such as dermatology journals linking to regenerative medicine and molecular biology outlets—underscore the field's reliance on cross‐disciplinary knowledge exchange. Collectively, these networks illustrate that PRP and skin aging research draws on, and contributes to, multiple academic domains, with core journals spanning both specialized and interdisciplinary platforms to drive knowledge dissemination and collaborative progress.

### 3.5 Authors

**FIGURE 6:**
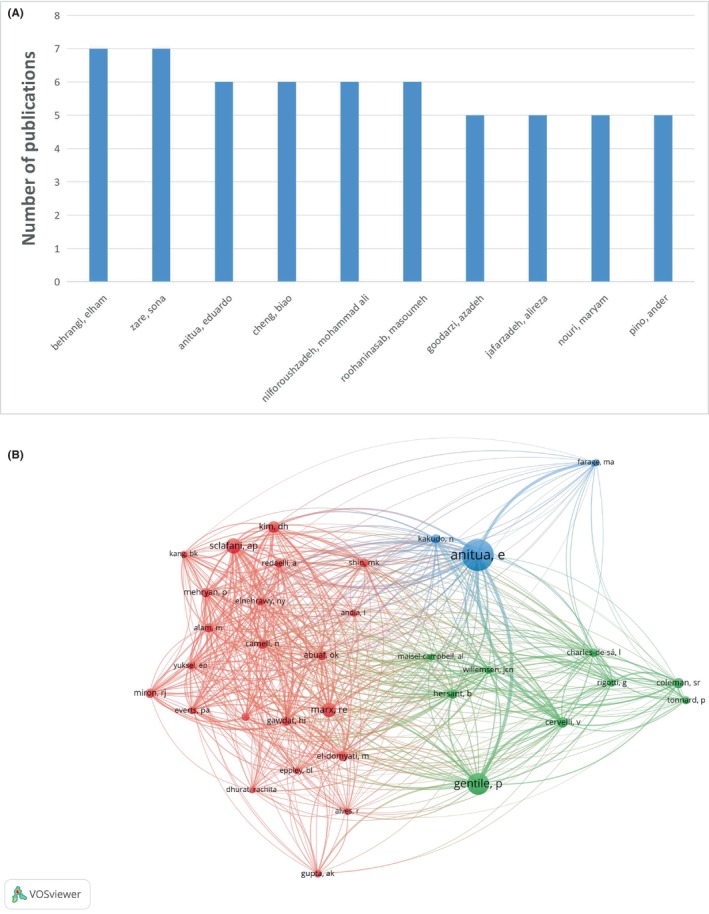
Authors and cocited authors analysis. (A) The top 10 productive authors. Each bar in blue represents the number of publications of each author. (B) Cocited authors analysis map. The size of the nodes represents the number of cocitations.

Figure 6A presents the publication output of the top 10 most productive authors: Behrangi, Elham, and Zare, Sona lead with 7 publications each, demonstrating their sustained contributions as core leaders in the field. Anitua, Eduardo, Cheng, Biao, Nilforoushzadeh, Mohammad Ali, and Roohaninasab, Masoumeh each contribute 6 publications, forming a productive second tier of researchers. Goodarzi, Azadeh, Jafarzadeh, Alireza, Nouri, Maryam, and Pino, Ander each have 5 publications, completing the top 10 list. Notably, Iranian researchers dominate this ranking, with 6 out of the top 10 authors affiliated with Iranian institutions, reflecting the country's emerging leadership in PRP–skin aging research. Collectively, these top authors drive a significant portion of the field's output, and the collaborative networks (Figure 6B) further demonstrate that team‐based and cross‐group partnerships are pivotal to advancing research on PRP and skin aging.

Figure 6B expands this visualization to show the author co‐authorship network. Anitua, E. emerges as a prominent central hub (the largest blue node), with extensive connections to researchers across multiple clusters, underscoring their leadership and collaborative influence in the field. The red cluster on the left features researchers such as Sclafani, A.P., Kim, D.H., and Kang, B.K., forming a tightly knit team focused on aesthetic and reconstructive applications, while the green cluster on the right includes Gentile, P., Cervelli, V., and Coleman, S.R., representing another active research group specializing in regenerative and surgical applications. Cross‐cluster links—such as those between Anitua, E. and researchers in both red and green clusters—illustrate the integration of diverse expertise and international collaboration in advancing PRP research.

### References

3.6

Among the retrieved publications on PRP and skin aging, the top 10 most co‐cited references (Table 4) exert profound academic influence, with each accumulating substantial citations ranging from 133 to 476. Collectively, these high‐impact studies form the theoretical cornerstone of the field, shaping core research directions such as PRP mechanisms in wound healing, hair regeneration, stem cell applications, and translational aesthetic interventions.

**TABLE 4:** The characteristics of highly cited and the most impactful classic articles in PRP in skin aging research field.

	Total citations	Article title	Journal	Published year	Country	IF2025
1	476	Exosomes from human umbilical cord blood accelerate cutaneous wound healing through miR‐21‐3p‐mediated promotion of angiogenesis and fibroblast function	THERANOSTICS	2018	China	13.3
2	395	Hopes and limits of adipose‐derived stem cells (ADSCs) and mesenchymal stem cells (MSCs) in wound healing	INTERNATIONAL JOURNAL OF MOLECULAR SCIENCES	2020	Morocco	4.9
3	240	The effect of platelet‐rich plasma in hair regrowth: a randomized placebo‐controlled trial	STEM CELLS TRANSLATIONAL MEDICINE	2015	Italy	4.9
4	178	Instructive microenvironments in skin wound healing: Biomaterials as signal releasing platforms	ADVANCED DRUG DELIVERY REVIEWS	2018	Spain	17.6
5	170	Effect of high‐volume injection, platelet‐rich plasma, and sham treatment in chronic midportion achilles tendinopathy a randomized double‐blinded prospective Study	AMERICAN JOURNAL OF SPORTS MEDICINE	2017	Denmark	4.5
6	167	Emerging treatment strategies in wound care	INTERNATIONAL WOUND JOURNAL	2022	Iran	2.5
7	166	Applications of mesenchymal stem cells in skin regeneration and rejuvenation	INTERNATIONAL JOURNAL OF MOLECULAR SCIENCES	2021	South Korea	4.9
8	164	Mesenchymal stem cells and cutaneous wound healing: current evidence and future potential	STEM CELLS INTERNATIONAL	2015	Ireland	3.3
9	146	Stem cells and exosomes: new therapies for intervertebral disc degeneration	CELLS	2021	USA	5.2
10	133	Mesenchymal stem cells from adipose tissue in clinical applications for dermatological indications and skin aging	INTERNATIONAL JOURNAL OF MOLECULAR SCIENCES	2017	USA	4.9

The most influential study, with 476 citations, is “Exosomes from human umbilical cord blood accelerate cutaneous wound healing through miR‐21‐3p‐mediated promotion of angiogenesis and fibroblast function” published in *Theranostics* (2018, IF2025 = 13.3) [12] by researchers from China, which established a critical foundation for understanding cell‐derived exosomes in skin repair. The second most cited work (395 citations), “Hopes and Limits of Adipose‐Derived Stem Cells (ADSCs) and Mesenchymal Stem Cells (MSCs) in Wound Healing” from the *International Journal of Molecular Sciences* (2020, Morocco) [13], provides comprehensive insights into the therapeutic potential of stem cells. Notably, the third‐ranked publication (240 citations), “The Effect of Platelet‐Rich Plasma in Hair Regrowth: A Randomized Placebo‐Controlled Trial” published in Stem Cells Translational Medicine (2015, Italy) [14], represents a landmark clinical study directly validating PRP efficacy in hair restoration. Other significant contributions include research on biomaterial‐based wound healing platforms (178 citations, *Advanced Drug Delivery Reviews*, Spain), PRP applications in Achilles tendinopathy (170 citations, *American Journal of Sports Medicine*, Denmark), and emerging treatment strategies in wound care (167 citations, *International Wound Journal*, Iran). The remaining top‐cited works focus on mesenchymal stem cells in skin regeneration and rejuvenation (166 and 133 citations), stem cells and exosomes in intervertebral disc degeneration (146 citations), and MSCs from adipose tissue for dermatological indications (133 citations)—collectively demonstrating the field's emphasis on regenerative medicine, cell‐based therapies, and PRP applications.

**FIGURE 7:**
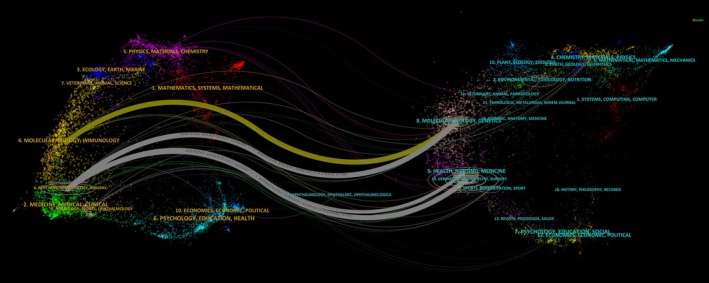
The dual‐map overlay of citing‐citation relationships among articles, with the citing journals on the left and the cited journals on the right. The colored path represents the citation relationship.

Regarding the dual‐map overlay of citation pathways (Figure 7), each dot represents a journal, while colored connecting curves quantify the strength and direction of citation links between citing journals (left panel) and cited journals (right panel). Two prominent pathways encapsulate the field's interdisciplinary knowledge exchange. First, research published in Dentistry, Dermatology, and Surgery journals is frequently cited in Molecular, Biology, and Genetics outlets (*z* = 4.57, *f* = 738)—a pattern that reflects the translation of clinical applications of PRP in aesthetic and reconstructive procedures into mechanistic frameworks for understanding platelet‐derived growth factors and tissue regeneration. Second, studies from Dentistry, Dermatology, and Surgery journals are widely cited in Health, Nursing, and Medicine publications (*z* = 4.70, *f* = 757), underscoring how clinical observations of PRP‐related skin and wound healing outcomes inform broader medical investigations of regenerative therapies and anti‐aging interventions. Additionally, a significant pathway connects Molecular, Biology, and Immunology research to Molecular, Biology, and Genetics outlets, highlighting the fundamental biological mechanisms underlying PRP action. Collectively, these pathways vividly illustrate the field's integration of clinical practice, basic science, and regenerative medicine, forging robust cross‐disciplinary bridges that advance both theoretical understanding and practical applications in PRP‐related skin aging research.

### 3.7 Keywords

**FIGURE 8:**
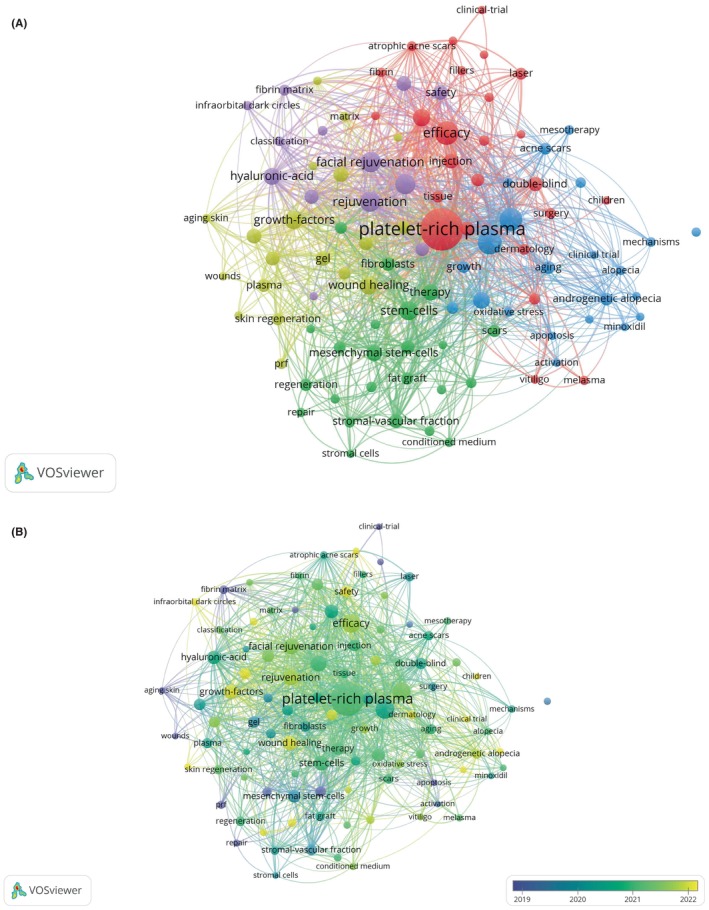
Keywords co‐occurrence analysis of PRP in skin aging research (A) Keywords co‐occurrence network. (The 102 keywords that appeared more than 5 times were selected. Cluster 1: red, cluster 2: green, cluster 3: blue, cluster 4: yellow; cluster 5: purple). (B) Keywords visual analysis according to the APY. (The color of the dot represents the time when the keyword appeared. The more recent the keyword, the more yellow the dot color).

To identify research hotspots, we performed a keyword co‐occurrence analysis using VOSviewer (Figure 8A). The network is centered on “platelet‐rich plasma,” linking core themes such as “efficacy” (and related terms like “safety,” “clinical trial,” and “double‐blind”), “rejuvenation” (“facial rejuvenation,” “injection”), and “wound healing” (“therapy,” “skin regeneration”). Other key clusters encompass aesthetic applications (“hyaluronic acid,” “fillers,” “laser,” “mesotherapy,” “acne scars”), regenerative mechanisms (“growth factors,” “fibroblasts,” “stem cells,” “mesenchymal stem cells”), clinical conditions (“androgenetic alopecia,” “alopecia,” “aging,” “scars,” “vitiligo,” “melasma”), and advanced therapies (“fat graft,” “stromal vascular fraction,” “conditioned medium,” “PRF”). This reflects the interdisciplinary integration of dermatology, aesthetic surgery, regenerative medicine, and cell biology.

Figure 8B color‐codes keywords by average year of appearance (yellow indicates more recent terms, ranging from 2019 to 2022). Established keywords (e.g., “platelet‐rich plasma,” “wound healing,” “growth factors,” “skin regeneration”) have an earlier average publication year (blue‐green nodes, around 2019–2020), forming the foundational framework of PRP research. In contrast, more recent keywords (yellow nodes, 2021–2022) include “efficacy,” “safety,” “clinical trial,” “double‐blind,” “mechanisms,” and “children”—highlighting emerging hotspots focused on rigorous clinical validation, evidence‐based practice, and expanded patient populations (including pediatric applications). Additionally, terms such as “oxidative stress,” “apoptosis,” and “activation” reflect growing interest in the cellular and molecular mechanisms underlying PRP action, signaling a shift toward mechanistic and translational research. Notably, keywords related to combination therapies (e.g., “hyaluronic acid,” “laser,” “fillers”) remain tightly connected to core terms, underscoring sustained interest in synergistic approaches in clinical practice.

## 4 Discussion

In this study, we retrieved 310 original articles and reviews published between 2014 and 2026 from the Web of Science Core Collection, focusing on PRP in skin aging. Using CiteSpace, VOSviewer, Tableau, and Excel, we systematically evaluated publication trends, geographic and institutional contributions, core journals, influential authors, citation patterns, and research hotspots.

The field has shown a marked upward trajectory, particularly since 2018, with 2025 recording the highest number of publications (47 papers). This growth reflects increasing recognition of PRP as a key therapeutic modality in skin aging intervention, driven by its roles in growth factor delivery, tissue regeneration, and extracellular matrix remodeling. The surge in publications suggests that the field is transitioning from preliminary exploration to rapid expansion, with growing interest in both basic mechanisms and translational applications. The moderate fluctuations in publication output, including temporary declines in 2019 and 2022, may reflect shifting research priorities, changes in funding landscapes, or the impact of global events such as the COVID‐19 pandemic on clinical research activities.

Geographically, the USA leads in publication output (79 papers), followed by China (54) and Italy (40), highlighting North America, East Asia, and Europe as major research hubs. The USA's dominance is further supported by the highest total citations (2328) and a strong citation‐per‐publication ratio (29.47). China's substantial contribution (17.66%) reflects its rapidly expanding capacity in regenerative medicine and aesthetic dermatology. Iran demonstrates emerging leadership with 4 institutions among the top 10 most productive, including Iran University of Medical Sciences ranking first (13 publications). However, the need to enhance research quality—such as through higher citation impact—remains, as evidenced by the superior citation performance of studies from the USA and European institutions, including the University of California San Diego (55.33 citations per paper) and IRCCS, Italy (36.8 citations per paper). International collaboration, as visualized in country and institutional networks, emerges as a key driver of progress, with the USA serving as a central hub connecting with China, Italy, and Brazil, while European nations form tightly linked cooperative clusters and Iranian institutions demonstrate strong internal partnerships with selective global linkages. Beyond publication outputs, clinical translation of PRP varies considerably across leading countries. In the United States, PRP is widely utilized off‐label for facial rejuvenation, androgenetic alopecia, and scar revision, though the FDA has not granted formal approval for aesthetic indications. In China, PRP has gained popularity for melasma, acne scars, and hair restoration, with consensus guidelines issued by the Chinese Medical Association, yet considerable heterogeneity in practice persists. In Italy, PRP is frequently combined with laser therapy and hyaluronic acid fillers, and national standards for hospital‐based PRP production ensure procedural uniformity. In Iran, despite a remarkable rise in research activity, PRP is increasingly applied for vitiligo, melasma, and chronic wounds, but limited access to advanced devices and varying regulatory oversight hinder clinical standardization. These country‐specific patterns underscore the gap between evidence generation and clinical implementation, particularly in regions where regulatory frameworks lag behind scientific progress. Future efforts should prioritize harmonizing preparation standards, conducting multicenter condition‐specific trials, and fostering regulatory alignment to ensure safe, effective, and equitable access to PRP‐based anti‐aging therapies worldwide.

In terms of publication venues, the *Journal of Cosmetic Dermatology* (42 articles) and *Aesthetic Surgery Journal* (13 articles) are prominent, reflecting the interdisciplinary nature of research across aesthetic dermatology and plastic surgery. Notably, *International Journal of Molecular Sciences* (IF 4.9) has the highest total citation count (718) despite moderate publication volume, and *Frontiers in Bioengineering and Biotechnology* (IF 4.8) also demonstrates strong citation performance, indicating their influence in translating PRP research into molecular and biotechnological insights. Journal co‐citation networks further illustrate integration across dermatology, regenerative medicine, and molecular biology, with high‐impact general science journals (e.g., Nature, Science, New England Journal of Medicine) serving as key connectors between clinical applications and fundamental mechanisms.

Influential studies, such as Hu L's 2018 article in *Theranostics* (476 citations) on exosome‐mediated wound healing and the landmark PRP hair regrowth trial by Gentile et al. (2015, 240 citations) in Stem Cells Translational Medicine, have provided foundational insights into growth factor and stem cell‐mediated mechanisms in skin biology. These studies have helped establish PRP as a viable therapeutic approach for skin regeneration and anti‐aging, guiding subsequent research into its clinical applications and mechanistic underpinnings. The dual‐map overlay of citation pathways further reveals robust interdisciplinary knowledge exchange, with clinical research in dentistry, dermatology, and surgery frequently cited in molecular biology, genetics, and broader medical outlets, underscoring the translation of PRP applications into mechanistic frameworks and evidence‐based practice.

Analysis of research hotspots reveals enduring themes such as “platelet‐rich plasma,” “skin rejuvenation,” “wound healing,” and “growth factors,” alongside emerging focuses including “efficacy,” “safety,” “clinical trial,” “double‐blind,” and “mechanisms.” The temporal distribution of keywords indicates a significant shift toward rigorous clinical validation and evidence‐based practice (2021–2022), marking the field's maturation from exploratory studies to randomized controlled trials and systematic investigations. Additionally, terms such as “oxidative stress,” “apoptosis,” and “activation” reflect growing attention to the cellular and molecular mechanisms underlying PRP action, while combination therapy keywords (“hyaluronic acid,” “laser,” “fillers,” “mesotherapy”) remain tightly connected to core themes, highlighting sustained interest in synergistic aesthetic modalities.

The author co‐authorship network identifies Anitua E. as a prominent central hub with extensive collaborative connections across multiple clusters, underscoring their leadership in advancing PRP research. Iranian researchers dominate the top 10 most productive authors (6 out of 10), including Behrangi E. and Zare S. (7 publications each), reflecting the country's concentrated expertise and emerging influence in this domain. Cross‐cluster collaborations between aesthetic‐focused researchers (e.g., Sclafani A.P., Kim D.H.) and regenerative medicine specialists (e.g., Gentile P., Cervelli V.) illustrate the integration of diverse expertise and international cooperation in driving field advancement.

This study has several limitations. First, it relies on a single database (Web of Science), which may exclude relevant publications from other sources. Second, non‐English literature was omitted, potentially introducing language bias. Third, recently published articles with low citation counts may be underrepresented. Fourth, the search was conducted on March 3, 2026, resulting in incomplete capture of 2026 publications (only 4 papers), which may affect trend analysis for the most recent year. Finally, the analysis does not account for publication quality beyond citation metrics, such as study design rigor or clinical outcome significance. Future bibliometric studies could integrate multiple databases (e.g., PubMed, Scopus) and longer timeframes to capture broader trends, while prospective studies should focus on high‐quality randomized controlled trials to establish definitive clinical efficacy and safety profiles of PRP in skin aging interventions.

## 5 Conclusion

In conclusion, this bibliometric analysis reveals that PRP and skin aging research has evolved into a mature, interdisciplinary field with sustained growth, global participation, and significant translational potential. The USA and China lead in research output and collaboration, while Iranian institutions and key researchers drive emerging contributions. Emerging hotspots such as clinical validation through randomized controlled trials, combination therapies with hyaluronic acid and laser, and oxidative stress mechanisms, coupled with the field's interdisciplinary knowledge exchange spanning dermatology, regenerative medicine, and molecular biology, highlight promising directions for future research. Strengthening international collaboration—particularly bridging regionally clustered networks, prioritizing research quality and evidence‐based translation, and expanding data sources—will be critical to advancing our understanding of PRP‐mediated skin aging and developing safe, effective interventions to promote healthy skin aging globally.

## Funding

The present study was supported by Zhejiang Provincial Health Commission Science and Technology Plan Project (2025HY1334) and Zhejiang Provincial Traditional Chinese Medicine Science and Technology Program (2026ZL0967).

## Ethics Statement

The authors have nothing to report.

## Conflicts of Interest

The authors declare no conflicts of interest.

## Data Availability

The data that support the findings of this study are available on request from the corresponding author. The data are not publicly available due to privacy or ethical restrictions.
